# The Army Medical Service

**Published:** 1905-11-04

**Authors:** 


### The Hospital.

A Journal of

The Medical Sciences and Hospital Administration.

Vol. XXXIX.—No. 997. SATURDAY, NOVEMBER 4, 1905.

### THE ARMY MEDICAL SERVICE.

The important discourse upon the Army Medical Service, delivered by Sir Frederick Treves before the Brighton and Sussex Medico-Chirurgical Society on October 27, is likely to exert an influence extending far beyond the audience to which it was addressed. Sir Frederick is able to judge of the medical requirements of the Army as a professional man; he has enjoyed exceptional opportunities of observing to what extent those requirements have hitherto been supplied, and he possesses unusual facilities for bringing his convictions to bear upon the minds of those most able to give effect to them. The officers of the Royal Army Medical Corps are said to be satisfied with the removal of the conditions which formerly deprived them of reasonable leave, and of reasonable opportunities of study; and the work of the officers will for the future be greatly facilitated by the aid to be derived from good nurses and effective and properly trained subordinates. As a general result, Sir Frederick declares that the Service is now second to none in the world, that it possesses a corps of highly qualified and most efficient medical officers, an up-to-date equipment, and a most carefully elaborated organisation; and that, as soon as projected extensive alterations in the hospitals are carried out, the sick soldier *in time of peace* will need nothing in the matters of method and treatment. The qualification which we have italicised reminds us of Kinglake's assertion that in Europe, during the first half of the nineteenth century, the end had been so much forgotten in pursuit of the means, and the industry exerted in the regulation of troops in peace time had become so foreign to the business of war, that the more a man was military in the narrow sense of the term, the less he was likely to be fitted for the perturbing exigencies of a campaign. In Russia this feeling had been carried so far that an army and war had actually been treated as things antagonistic the one to the other, and that the Grand Duke Constantine once declared that he dreaded a war, because he was sure that it would spoil the troops which, with ceaseless care and labour, he had striven to bring to perfection.

We will not accuse Sir Frederick Treves, or any other Englishman, of "dreading" a war, however much he might for good reasons be averse to it; but still it is not difficult, if we read between the lines of his discourse, to perceive that he has no confidence in the adaptability for war of the organisation which he has described. Its chief defects appear to be that it is under the supreme control of a non-medical official, who has the power of the purse, and would necessarily be unable, from sheer ignorance, to anticipate the wants of the Service, even if he were capable of understanding them when they were explained to him. Sir Frederick states it to be essential that the Director-General of the Medical Service should be the head of his department, and responsible for its efficiency and economical administration; that he should have direct access to the Army Council and to the Secretary of State for War; and that he should have control of the money voted for the medical service. He laid stress upon the fact that the great bulk of the casualties in war are due to preventible disease, which, if it were preventible should be prevented; while the power of the army doctor to do this is practically *nil*. In a recent letter to the *Times* on the subject of army typhoid, and in opposition to the proposals for the systematic boiling of drinking water put forward by Dr. Leigh Canney, Surgeon-General Gallwey disputed the general belief that the typhoid at Bloemfontein was due to the contaminated water at Paardeberg, and attributed it to the "grime, dust, dirt of war, which harbour the bacilli on the human being, on his clothes, on his bedding, on his food, while the overcrowding of tents and ground spreads the disease by contact and by lowering vitality." It is, of course, impossible to exclude the conditions which Surgeon-General Gallwey thus describes, and equally impossible to form any precise estimate of the degree of influence which they may exert; but common sense points to water pollution as the main channel of diffusion of the disease, and it is satisfactory to obtain from Sir Frederick, so soon after the publication of Surgeon-General Gallwey's antagonistic views, the precise and simple declaration that "the casualties at Colenso were as nothing to those sustained through the men drinking the contaminated water of the Modder river when peacefully crossing it on a perfectly calm day." It may be regarded as certain that disease-prevention will never attain its full possibilities in campaigning until the importance of the subject is better appreciated than at present by our "military" authorities, who have no knowledge either of the ravages of disease or of the requirements of war. Sir Frederick rightly lays it down, among conditions essential to the attainment of complete efficiency in the field, that the combatant officer should have some knowledge of hygiene as applied to campaigning and barrack life, that a like knowledge, of a more elementary character, should be possessed by the private soldier, and that the Army medical officer should be vested with such authority and provided with such *personnel* as would enable him to carry out those sanitary arrangements in the field which experience has proved to be absolutely essential to secure the minimum loss of life from disease. To these requirements he adds that of an effective medical reserve to be called out in time of war; but this branch of the subject is too large, and too important, to be taken into consideration at the close of an article.

